# Supplementary material for: The somatic mutation landscape of normal gastric epithelium
Source: Nature. 2025 Mar 19;640(8058):418–26. doi: 10.1038/s41586-025-08708-6 (PMC11981919; doi:10.1038/s41586-025-08708-6)
Supplement: Supplementary file 2 — Reporting Summary [file 41586_2025_8708_MOESM2_ESM.pdf]

nature portfolio

Corresponding author(s): Tim Coorens, SY Leung, Mike Stratton

Last updated by author(s): Jan 20, 2025

Reporting Summary

Nature Portfolio wishes to improve the reproducibility of the work that we publish. This form provides structure for consistency and transparency in reporting. For further information on Nature Portfolio policies, see our Editorial Policies and the Editorial Policy Checklist.

Statistics

For all statistical analyses, confirm that the following items are present in the figure legend, table legend, main text, or Methods section.

n/a	Confirmed	
☐	☒	The exact sample size (*n*) for each experimental group/condition, given as a discrete number and unit of measurement
☐	☒	A statement on whether measurements were taken from distinct samples or whether the same sample was measured repeatedly
☐	☒	The statistical test(s) used AND whether they are one- or two-sided<br>*Only common tests should be described solely by name; describe more complex techniques in the Methods section.*
☐	☒	A description of all covariates tested
☐	☒	A description of any assumptions or corrections, such as tests of normality and adjustment for multiple comparisons
☐	☒	A full description of the statistical parameters including central tendency (e.g. means) or other basic estimates (e.g. regression coefficient) AND variation (e.g. standard deviation) or associated estimates of uncertainty (e.g. confidence intervals)
☐	☒	For null hypothesis testing, the test statistic (e.g. *F*, *t*, *r*) with confidence intervals, effect sizes, degrees of freedom and *P* value noted<br>*Give P values as exact values whenever suitable.*
☐	☒	For Bayesian analysis, information on the choice of priors and Markov chain Monte Carlo settings
☒	☐	For hierarchical and complex designs, identification of the appropriate level for tests and full reporting of outcomes
☒	☐	Estimates of effect sizes (e.g. Cohen's *d*, Pearson's *r*), indicating how they were calculated

Our web collection on statistics for biologists contains articles on many of the points above.

Software and code

Policy information about availability of computer code

Data collection: No software was used for data collection.

Data analysis:
- Alignment: BWA (https://github.com/lh3/bwa) (v0.7.17)
- SNV variant calling: CaVEMan (https://github.com/cancerit/CaVEMan) (v.1.14.0)
- Indel calling: Pindel (https://github.com/cancerit/cgpPindel) (v.3.9.0)
- CNV calling: ASCAT (https://github.com/cancerit/ascatNgs) (v.4.4.1)
- SV calling: GRIDDS (https://github.com/PapenfussLab/gridss) (v.2.13.2)
- Mutational signature analysis: HDP (https://github.com/nicolaroberts/hdp (v1)
- Mutational signature analysis: SigFit (https://github.com/kgori/sigfit) (v2.0.0)
- Phylogeny reconstruction: Sequoia (https://github.com/TimCoorens/Sequoia) (v1)
- Phylogeny reconstruction: MPBoot (https://github.com/diepthihoang/mpboot) (v1)
- Mutation mapping: treemut (https://github.com/NickWilliamsSanger/treemut) (v1)
- Telomere length estimation: TelomereCat (https://github.com/cancerit/telomerecat) (v4.0.1)

Custom R scripts for data analysis, filtering and visualization can be found at https://github.com/TimCoorens/Stomach

For manuscripts utilizing custom algorithms or software that are central to the research but not yet described in published literature, software must be made available to editors and reviewers. We strongly encourage code deposition in a community repository (e.g. GitHub). See the Nature Portfolio guidelines for submitting code & software for further information.

Data

Policy information about availability of data

All manuscripts must include a data availability statement. This statement should provide the following information, where applicable:

- Accession codes, unique identifiers, or web links for publicly available datasets
- A description of any restrictions on data availability
- For clinical datasets or third party data, please ensure that the statement adheres to our policy

DNA sequencing data have been deposited in the European Genome-Phenome Archive (EGA) with accession codes EGAD00001015351 (whole-genome sequencing) and EGAD00001015352 (targeted panel sequencing). Processed data are available in the Supplementary Tables or on GitHub (https://github.com/TimCoorens/Stomach; filtered variant calls and phylogenies). Reference genome GRCh38 is widely available (including at https://www.ncbi.nlm.nih.gov/datasets/genome/GCF_000001405.26/).

Research involving human participants, their data, or biological material

Policy information about studies with human participants or human data. See also policy information about sex, gender (identity/presentation), and sexual orientation and race, ethnicity and racism.

Reporting on sex and gender	The study includes 12 donors of the female sex and 18 donors of the male sex.
Reporting on race, ethnicity, or other socially relevant groupings	The study includes 11 donors from Hong Kong, all of whom report South-East Asian ethnicity, and 3 donors from the UK and 16 from the US, all of whom are reported as "White" or "Caucasian" ethnicity.
Population characteristics	Data was obtained from 18 gastric cancer patients and 12 non-cancer donors. These donors span the US (n=16), UK (n=3) and Hong Kong (n=11), and are of various ages (between age 23 and 85)
Recruitment	1. Multi-site sampling was performed on gastrectomy specimens removed either as part of gastric cancer treatment or bariatric surgery (Hong Kong University)<br>2. Multi-region gastric biopsies from transplant organ donors with informed consent for participation in research obtained from the donor’s family as part of the Cambridge Biorepository for Translational Medicine program (UK)<br>3. Gastric samples obtained at autopsy from AmsBio (commercial supplier) (US)
Ethics oversight	Snap-frozen gastric biopsy samples were obtained from three sources:<br>1. Multi-site sampling was performed on gastrectomy specimens removed either as part of gastric cancer treatment or bariatric surgery. Written informed consent for participation in research was obtained from all donors in accordance with the Declaration of Helsinki and protocols approved by the relevant research ethics committees (RECs): (i) source country approval by the IRB of the University of Hong Kong/Hospital Authority of Hong Kong West Cluster, REC approval reference number UW14-257; (ii) UK NHS REC approval from the West Midlands-Coventry and Warwickshire REC, approval number 17/WM/0295, UK Integrated Research Application System (IRAS) project ID 228343.<br>2. Multi-region gastric biopsies from transplant organ donors with informed consent for participation in research obtained from the donor’s family as part of the Cambridge Biorepository for Translational Medicine program (UK NHS REC approval reference number 15/EE/0152; approved by NRES Committee East of England – Cambridge South).<br>3. Gastric samples obtained at autopsy from AmsBio (commercial supplier). UK NHS REC approving the use of these samples: London-Surrey Research Ethics Committee, REC approval reference number 17/LO/1801.

Note that full information on the approval of the study protocol must also be provided in the manuscript.

Field-specific reporting

Please select the one below that is the best fit for your research. If you are not sure, read the appropriate sections before making your selection.

☒ Life sciences ☐ Behavioural & social sciences ☐ Ecological, evolutionary & environmental sciences

For a reference copy of the document with all sections, see nature.com/documents/nr-reporting-summary-flat.pdf

Life sciences study design

All studies must disclose on these points even when the disclosure is negative.

Sample size	The cohort consists of 30 individuals, 18 with gastric cancer and 12 with no gastric pathology, from Hong Kong, the United States or the United Kingdom. From these donors, 217 normal gastric glands and 21 neoplastic glands from the gastric cancers of two individuals were microdissected and individually whole genome sequenced to 23-fold median coverage. In addition, we subjected a further 829 microdissections comprising a total of 8,0007 gastric glands to targeted sequencing of known cancer genes. The numbers of individuals and glands sampled per individual are in line with previous efforts to map somatic mutation landscapes in colon (Lee-Six et al, 2019, Nature), endometrium (Moore et al., 2020, Nature) and placenta (Coorens et al., 2021, Nature) and so the sample sizes were deemed sufficient to achieve the aims of the study.

Data exclusions	Data with poor coverage after laser capture microdissection and whole-genome sequencing (< 10x) were excluded from analysis.
Replication	The LCM and sequencing pipeline, as well as the variant calling and data analysis pipelines, have been extensively replicated and validated across more than 10 studies. (Ellis et al., 2021, Nature Protocols; Moore et al., 2021, Nature; Brunner et al. ,2019, Nature; Lee-Six et al., 2019, Nature; Coorens et al. 2021, Nature; Lawson et al., 2020, Science; Moore et al., 2020, Nature; Robinson et al., 2021, Nature Genetics; Olafsson et al., 2020, Cell; Wang et al., 2022, Nature Genetics).
Randomization	Not applicable to this study - donor status as cancer patient or non-cancer donor was known a priori. Study describes the landscape of somatic mutations in gastric epithelium across individuals and hence is descriptive in nature, rather than testing a specific hypothesis in the population (which may have required randomization).
Blinding	Not applicable to this study - donor status as cancer patient or non-cancer donor was known a priori.Study describes the landscape of somatic mutations in gastric epithelium across individuals and hence is descriptive in nature, rather than testing a specific hypothesis in the population (which may have required blinding).

Reporting for specific materials, systems and methods

We require information from authors about some types of materials, experimental systems and methods used in many studies. Here, indicate whether each material, system or method listed is relevant to your study. If you are not sure if a list item applies to your research, read the appropriate section before selecting a response.

Materials & experimental systems

n/a	Involved in the study
☒	☐ Antibodies
☒	☐ Eukaryotic cell lines
☒	☐ Palaeontology and archaeology
☒	☐ Animals and other organisms
☒	☐ Clinical data
☒	☐ Dual use research of concern
☒	☐ Plants

Methods

n/a	Involved in the study
☒	☐ ChIP-seq
☒	☐ Flow cytometry
☒	☐ MRI-based neuroimaging

Plants

Seed stocks	*Report on the source of all seed stocks or other plant material used. If applicable, state the seed stock centre and catalogue number. If plant specimens were collected from the field, describe the collection location, date and sampling procedures.*
Novel plant genotypes	*Describe the methods by which all novel plant genotypes were produced. This includes those generated by transgenic approaches, gene editing, chemical/radiation-based mutagenesis and hybridization. For transgenic lines, describe the transformation method, the number of independent lines analyzed and the generation upon which experiments were performed. For gene-edited lines, describe the editor used, the endogenous sequence targeted for editing, the targeting guide RNA sequence (if applicable) and how the editor was applied.*
Authentication	*Describe any authentication procedures for each seed stock used or novel genotype generated. Describe any experiments used to assess the effect of a mutation and, where applicable, how potential secondary effects (e.g. second site T-DNA insertions, mosiacism, off-target gene editing) were examined.*
